# A Bead-based Normalization for Uniform Sequencing depth (BeNUS) protocol for multi-samples sequencing exemplified by *HLA-B*

**DOI:** 10.1186/1471-2164-15-645

**Published:** 2014-08-04

**Authors:** Kazuyoshi Hosomichi, Shigeki Mitsunaga, Hideki Nagasaki, Ituro Inoue

**Affiliations:** Division of Human Genetics, National Institute of Genetics, 1111 Yata, Mishima, 411-8540 Shizuoka, Japan; Department of Molecular Life Sciences, Tokai University School of Medicine, 143 Shimokasuya, 259-1143 Isehara, Kanagawa Japan; Genome Informatics Laboratory, Center for Information Biology and DNA Data Bank of Japan, National Institute of Genetics, 1111 Yata, Mishima, 411-8540 Shizuoka Japan

**Keywords:** HLA, Next-generation sequencing

## Abstract

**Background:**

Human leukocyte antigen (HLA) is a group of genes that are extremely polymorphic among individuals and populations and have been associated with more than 100 different diseases and adverse drug effects. HLA typing is accordingly an important tool in clinical application, medical research, and population genetics. We have previously developed a phase-defined HLA gene sequencing method using MiSeq sequencing.

**Results:**

Here we report a simple, high-throughput, and cost-effective sequencing method that includes normalized library preparation and adjustment of DNA molar concentration. We applied long-range PCR to amplify *HLA-B* for 96 samples followed by transposase-based library construction and multiplex sequencing with the MiSeq sequencer. After sequencing, we observed low variation in read percentages (0.2% to 1.55%) among the 96 demultiplexed samples. On this basis, all the samples were amenable to haplotype phasing using our phase-defined sequencing method. In our study, a sequencing depth of 800x was necessary and sufficient to achieve full phasing of *HLA-B* alleles with reliable assignment of the allelic sequence to the 8 digit level.

**Conclusions:**

Our HLA sequencing method optimized for 96 multiplexing samples is highly time effective and cost effective and is especially suitable for automated multi-sample library preparation and sequencing.

**Electronic supplementary material:**

The online version of this article (doi:10.1186/1471-2164-15-645) contains supplementary material, which is available to authorized users.

## Background

To date, several high-throughput HLA typing methods using next-generation sequencing (NGS) have been developed [1–8]. In our previous study, we completely sequenced long-range PCR amplicons encompassing entire regions of each of the HLA genes (*HLA-A, -C, -B, -DRB1, -DQB1,* and *-DPB1*). PCR amplicons were subjected to transposase-based library construction and multiplex sequencing with the MiSeq sequencer. Paired-end reads of 2 × 300 bp enabled us to demonstrate phase-defined allele determination (also defined as HLA gene haplotypes) in 33 HLA homozygous samples, 11 HLA heterozygous samples, and 3 parent–child families. Our sequencing protocol and pipeline provided essentially complete phase-defined HLA gene sequences; however, it required complicated and labor-intensive workflows especially in the library preparation step. Most importantly, the method is not well adapted for processing multiple samples. In the present study, we developed a new library preparation method for NGS and applied it to 96 samples. Long-range PCR products of *HLA-B* spanning from promoters to 3′-UTRs were prepared and sequenced with the MiSeq sequencer via transposase-based library preparation. In the previous protocol, although the DNA amount of each library was strictly measured and the library size was validated using a BioAnalyzer before the sequencing step, it was difficult to equalize the DNA amount and molecular size of the libraries, resulting in variable numbers of reads in each sample. We observed dropouts of samples owing to insufficient reads. Here, we developed a **Be**ad-based **N**ormalization for **U**niform **S**equencing (BeNUS) procedure using three steps of bead purification. BeNUS can easily and precisely normalize the molar concentrations of up to 96 samples, not only simplifying the library preparation step but also permitting automation of HLA typing using NGS.

## Results and discussion

### PCR amplification of *HLA-B*and library preparation

We applied long-range PCR to amplify *HLA-B*, which is known to be highly polymorphic. *HLA-B*-specific amplification products were obtained from 96 individuals. All 96 PCR amplicons were subjected to transposase-based library construction using the Nextera kit, which simultaneously fragments the DNA and adds adaptors needed for multiplex sequencing. We developed protocol steps using altered AMPure XP beads to normalize the molar concentrations of 96 samples. Each PCR amplicon was subjected separately to transposase-based library construction, whereby a sample-specific index was introduced. The Nextera kit can construct libraries of a broad size range. For phase-defined HLA sequencing, a library range of 500–1,000 bp is desirable. In the previous protocol [1], the library size selection was achieved by cutting of agarose gel and checking using a BioAnalyzer, which is a very laborious step, especially for preparing multiple samples.

**Figure 1:**
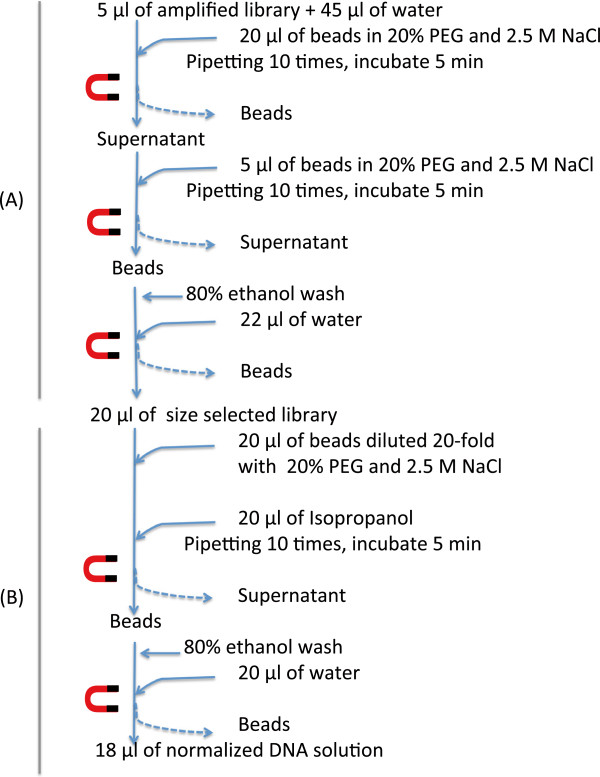
**Schematic workflow of BeNUS.** The BeNUS method was constructed using two categories of method, size selection **(A)** and normalization of DNA amount **(B)**. **(A)** Size selection using altered AMPure XP beads. For size selection, two different bead ratio conditions were applied according to DNA volume: 0.4× bead ratio for <1,000 bp fragment size and 0.5× bead ratio for >500 bp. After bead selection, DNA fragments ranging from 500 to 1,000 bp were bound to the beads. **(B)** Normalization of DNA amount using altered AMPure XP beads DNA fragments with target sizes ranging from 500 to 1,000 bp were selected for effective HLA gene haplotype phasing. The size selection and DNA amount also defined an actual molar concentration for bridge PCR to generate clusters in a flow cell, because DNA fragments of over 1,000 bp are not efficiently amplified. Only one bead reaction condition was applied to normalize the amount of DNA. This step enables a defined amount of DNA to be bound to the diluted beads. This step enables a defined amount of DNA to be bound to the diluted beads.

Our new method, BeNUS, which is described in detail in the Methods section, circumvents the gel cutting method and employs bead-based steps for library size selection as well as equalization of DNA molar concentrations in up to 96 samples. More specifically, three bead steps were performed: first, 20 μl of altered beads suspended in 20% PEG and 2.5 M NaCl solution was added to 50 μl of diluted PCR product, and the supernatant fraction containing the desired fragments (<1,000 bp) was collected. Second, 5 μl of beads was added to the collected supernatant. Desired fragments of larger than 500 bp were bound to the beads, while smaller fragments remaining in the supernatant were discarded (Figure 1). After these two steps, the desired DNA fragments (500–1,000 bp) were selected (actual size: 492 to 1,625 bp, average size: 924 bp) (Additional file 1: Figure S1). Finally, 20-fold diluted beads were added to the size-fractionated library. Small numbers of beads can bind saturated amounts of DNA because bound DNA is in proportion to the number of beads (Additional file 2: Figure S2). Eventually, the final DNA size, concentration, and thereby molar concentration were equalized (Additional file 3: Figure S3).

### Complete sequencing of *HLA-B*for 96 samples

**Figure 2:**
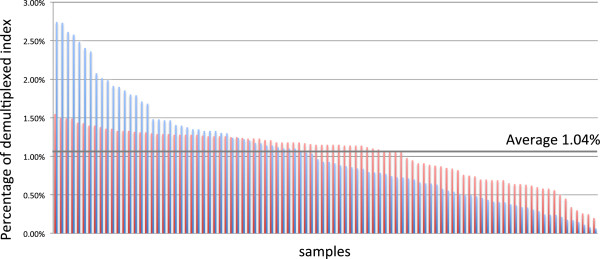
**Percentage of read numbers among 96 multiplexing indexes.** The horizontal axis shows the 96 samples and the vertical axis shows read percentage among demultiplexed 96 FASTQ files. Red bars show distribution of read number in proportion to libraries prepared by BioAnalyzer and agarose gel size selection, and blue bars show result by BeNUS method. Sequence reads for each sample were counted for evaluation of the normalization step in library preparation.

**Figure 3:**
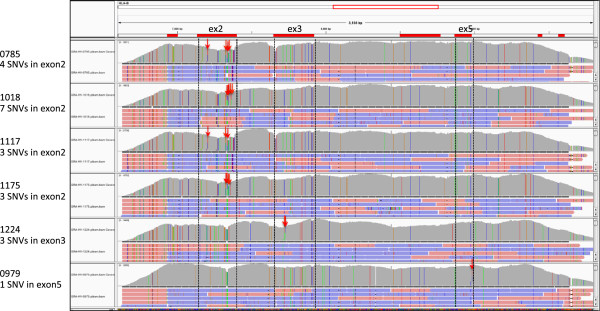
**Alignment view of heterozygous samples showing several SNVs or a single SNV between two HLA allele sequences in one exon.** The horizontal axis shows the position in *HLA-B* and red boxes at the top are exon regions. The vertical axis of upper graph in each sample shows read depth at each position, and red and blue bars in lower region are aligned reads as read1 (red) and read2 (blue). In the read depth graph, the gray color denotes bases identical with the reference genome, and, green, blue, orange, and red colors denote bases different from reference genome as A, C, G and T, respectively. If a position has 2 colors, it means heterozygous SNV, meanwhile, 1 color at one position means homozygous SNV. Red arrow indicates positions of the heterozygous SNVs.

The Nextera-treated libraries from 96 *HLA-B* PCR amplicons were subjected to NGS sequencing. The distribution of reads among the 96 multiplexed indexes ranged from 0.2% to 1.55% as percentages of 24.6 million reads and 1.04 Â± 0.32% on average. Technical improvements in libraries preparation were clarified by comparison between our previous method, cutting of agarose gel followed by the BioAnalyzer, and BeNUS method. The distribution of respective read numbers by the previous method was 0.06% to 2.74% and the value by BeNUS method was 0.2 to 1.55% (Figure 2). Sequence reads of *HLA-B,* were aligned to the reference sequence at an average mapped rate of 99.63%, ranging from 99.11 to 99.87%. The average depth of the alignment before phasing was 6495.3× and the phased haplotype depth was 2097.8×. Of the 96 samples, heterozygous SNVs and indels were not observed in 8 samples (0605, 0607, 0645, 1057, 1169, 1184, 1199, and 1229) indicating completely homozygous haplotypes. Generally, MiSeq yields base errors at a rate of 0.8% with PCR free library preparation [9]. In the result of these homozygous samples, the total error rate of sequencing reaction and PCR amplification was estimated to be as less than 1.2%. In our simulated alignment result, the maximum error rates in 300× and 200× average depth were 4.8% and 6%, respectively, and the minimum depth for complete phasing was approximately 800× average depth. We recognize phasing result is not only dependent on average depth but also on distance between two heterozygous SNVs and long insert library covering the two heterozygous SNVs. As a general method, we propose that sequence reads of at least 800× depth is valid for providing the phase-defined HLA sequences with for high accuracy (less than 5% error rate). All samples were completely phased by the phase-defined sequencing pipeline [1], although six samples showed only 1 to 7 SNVs in one exon: samples 0785, 1018, 1117, and 1175 had 4, 7, 3, and 3 heterozygous SNVs in exon 2, respectively. Sample 1224 had 3 heterozygous SNVs in exon 3, whereas sample 0979 had one heterozygous SNV in exon 5 (Figure 3). All the above samples were also phased using partial phasing (Additional file 4: Figure S4). Allelic imbalance as a result of PCR is manifested by skewed allelic calls after HLA sequencing. Allelic imbalance of the PCR amplification of *HLA-B* was negligible, as judged by the ratio of sequencing depth between the two phased alignments; 1:1.68 at the maximum and 1:1.19 on average in heterozygous samples (Table 1). Consequently, 192 haplotype sequences of 96 individuals, which include 28 different haplotype sequences, were constructed as phased allelic *HLA-B* sequences. In general, allelic imbalance after PCR amplification has been occasionally observed. Evidently, this problem is not specific to NGS analyses. If capture, NGS, or the analytical step are causes of allelic imbalance, we would observe the discrepancy between our NGS typing and SSO typing, which was not the case in the current study. The current protocol aimed at minimizing the disparity of each sample; this was achieved although not perfectly. The minimum depth could be important to obtain phase-defined sequences because lower depth could leave unphased region. However, complete phase-defined sequencing is dependent on the allelic type of the sample, thereby it is not easy to give an exact depth number to accomplish the sequencing in general. In the current study, the most important point of the current study was to obtain complete phase-defined sequences of 96 samples without any dropout, which was achieved using the current protocol.

**Table 1:** **Alignment of the** ***HLA-B*** **sequence and genotype in 96 samples**

Sample	HLA-B SSO		HLA-B PSP*		Depth		Note
	Allele 1	Allele 2	Allele 1	Allele 2	Allele 1	Allele 2	
0554	HLA-B*44:03	HLA-B*40:06	HLA-B*44:03:01:01	HLA-B*40:06:01:01	1543.4	1114.5	
0555	HLA-B*40:01	HLA-B*44:03	HLA-B*40:01:02	HLA-B*44:03:01	2228.5	2084.8	
0556	HLA-B*13:01	HLA-B*46:01	HLA-B*13:01:01	HLA-B*46:01:01	860.5	765	
0560	HLA-B*13:01	HLA-B*51:01	HLA-B*13:01:01	HLA-B*51:01:01	1416.5	1455.9	
0605	HLA-B*52:01	HLA-B*52:01	HLA-B*52:01:01:02	HLA-B*52:01:01:02	6928.7		Homozygous
0607	HLA-B*07:02	HLA-B*07:02	HLA-B*07:02:01	HLA-B*07:02:01	7357.3		Homozygous
0616	HLA-B*40:02	HLA-B*52:01	HLA-B*40:02:01	HLA-B*52:01:01:02	513.2	500.6	
0639	HLA-B*40:06	HLA-B*35:01	HLA-B*40:06:01:01	HLA-B*35:01:01:02	1758	1656.8	
0642	HLA-B*40:01	HLA-B*35:01	HLA-B*40:01:02	HLA-B*35:01:01:02	2016.8	1545.3	
0645	HLA-B*54:01	HLA-B*54:01	HLA-B*54:01:01	HLA-B*54:01:01	6537		Homozygous
0649	HLA-B*55: 02	HLA-B*40:02	HLA-B*55:02:01	HLA-B*40:02:01	1402.3	1635.5	
0652	HLA-B*15:01	HLA-B*35:01	HLA-B*15:01:01:01	HLA-B*35:01:01:02	2395.4	2308.4	
0658	HLA-B*56:01	HLA-B*44:03	HLA-B*56:01:01	HLA-B*44:03:01	1686.7	2074	
0663	HLA-B*13:01	HLA-B*52:01	HLA-B*13:01:01	HLA-B*52:01:01:02	1538.1	1455.7	
0666	HLA-B*54:01	HLA-B*38:02	HLA-B*54:01:01	HLA-B*38:02:01	2105	1790.8	
0703	HLA-B*55:02	HLA-B*40:06	HLA-B*55:02:01	HLA-B*40:06:01:01	1173.1	1445.3	
0735	HLA-B*44:03	HLA-B*38:02	HLA-B*44:03:01	HLA-B*38:02:01	2507.5	1998.5	
0739	HLA-B*40:02	HLA-B*48:01	HLA-B*40:02:01	HLA-B*48:01:01	1947.1	2382.9	
0741	HLA-B*59:01	HLA-B*07:02	HLA-B*59:01:01:01	HLA-B*07:02:01	1458.6	2336	
0772	HLA-B*07:02	HLA-B*48:01	HLA-B*07:02:01	HLA-B*48:01:01	3512.9	3425	
0779	HLA-B*44:02	HLA-B*39:01	HLA-B*44:02:01:01	HLA-B*39:01:01:03	1677.5	1060.6	
0784	HLA-B*40:06	HLA-B*15:01	HLA-B*40:06:01:01	HLA-B*15:01:01:01	1875.1	1820.8	
0785	HLA-B*52:01	HLA-B*51:01	HLA-B*52:01:01:01	HLA-B*51:01:01	6041.4	6018.4	Onlu 4 heterozygous SNVs
0810	HLA-B*40:02	HLA-B*46:01	HLA-B*40:02:01	HLA-B*46:01:01	560.8	546.8	
0815	HLA-B*35:01	HLA-B*07:02	HLA-B*35:01:01	HLA-B*07:02:01	1239.5	1740	
0821	HLA-B*40:01	HLA-B*40:02	HLA-B*40:01:02	HLA-B*07:02:01	1239.5	1740	
0821	HLA-B*40:01	HLA-B*40:02	HLA-B*40:01:02	HLA-B*40:02:01	2144.4	1925.5	
0822	HLA-B*54:01	HLA-B*35:01	HLA-B*54:01:01	HLA-B*35:01:01	1550	1840.6	
0823	HLA-B*15:11	HLA-B*07:02	HLA-B*15:11:01	HLA-B*07:02:01	703.5	914.6	
0830	HLA-B*51:01	HLA-B*48:01	HLA-B*51:01:01	HLA-B*48:01:01	1833.8	2509	
0969	HLA-B*40:06	HLA-B*51:01	HLA-B*40:06:01:01	HLA-B*51:01:01	1575.4	1650.4	
0975	HLA-B*44:03	HLA-B*07:02	HLA-B*44:03:01	HLA-B*07:02:01	180.4	209.3	
0976	HLA-B*54:01	HLA-B*35:01	HLA-B*54:01:01	HLA-B*35:01:01	3635.9	3759.2	
0977	HLA-B*44:02	HLA-B*15:18	HLA-B*44:02:01:01	HLA-B*15:18:01	722.2	574.4	
0979	HLA-B*39:01	HLA-B*39:01	HLA-B*39:01:01:03	HLA-B*39:01:03	2128	2125.7	Only 1 heterozygous SNV
0991	HLA-B*15:01	HLA-B*39:01	HLA-B*15:01:01	HLA-B*39:01:01:03	1685.4	1551.3	
0997	HLA-B*15:01	HLA-B*51:01	HLA-B*15:01:01:01	HLA-B*39:01:01:03	1685.4	1551.3	
0997	HLA-B*15:01	HLA-B*51:01	HLA-B*15:01:01	HLA-B*51:01:01	2588	2692.1	
0999	HLA-B*48:01	HLA-B*39:01	HLA-B*48:01:01	HLA-B*39:01:01:03	1470.4	1045.3	
1009	HLA-B*54:01	HLA-B*39:01	HLA-B*54:01:01	HLA-B*39:01:01:03	326.8	294.7	
1011	HLA-B*52:01	HLA-B*07:02	HLA-B*52:01:01:02	HLA-B*07:02:01	730.9	943.7	
1012	HLA-B*35:01	HLA-B*48:01	HLA-B*35:01:01:02	HLA-B*48:01:01	1830.1	2482.1	
1013	HLA-B*54:01	HLA-B*52:01	HLA-B*54:01:01	HLA-B*52:01:01:02	533.9	600.7	
1014	HLA-B*55:02	HLA-B*51:01	HLA-B*55:02:01	HLA-B*51:01:01	970.1	1153.1	
1016	HLA-B*44:03	HLA-B*35:01	HLA-B*44:03:01	HLA-B*35:01:01	739.7	655.5	
1018	HLA-B*46:01	HLA-B*15:01	HLA-B*46:01:01	HLA-B*15:01:01:01	5360.8	5496.1	Only 7 heterozygous SNVs
1030	HLA-B*40:06	HLA-B*07:02	HLA-B*40:06:01:01	HLA-B*07:02:01	1638.8	2668.6	
1045	HLA-B*15:11	HLA-B*35:01	HLA-B*15:11:01	HLA-B*35:01:01:02	1597.5	1448.8	
1056	HLA-B*58:01	HLA-B*67:01	HLA-B*58:01:01	HLA-B*67:01:01	2497.9	2214.5	
1057	HLA-B*40:02	HLA-B*40:02	HLA-B*40:02:01	HLA-B*40:02:01	4113.6		Homozygous
1064	HLA-B*40:06	HLA-B*51:01	HLA-B*40:06:01:01	HLA-B*51:01:01	963.5	1048.8	
1065	HLA-B*54:01	HLA-B*52:01	HLA-B*54:01:01	HLA-B*52:01:01:02	1213.9	1563.8	
1071	HLA-B*56:01	HLA-B*40:06	HLA-B*56:01:01	HLA-B*40:06:01:01	4061.2	1223	
1079	HLA-B*40:02	HLA-B*39:01	HLA-B*40:02:01	HLA-B*39:01:03	2319.8	1507.5	
1082	HLA-B*40:01	HLA-B*51:01	HLA-B*40:01:02	HLA-B*51:01:01	639.3	542.7	
1083	HLA-B*59:01	HLA-B*54:01	HLA-B*59:01:01	HLA-B*54:01:01	716.5	1208.2	
1087	HLA-B*44:03	HLA-B*07:02	HLA-B*44:03:01	HLA-B*07;02:01	1975.5	2605.1	
1095	HLA-B*40:02	HLA-B*54:01	HLA-B*40:02:01	HLA-B*51:01:01	946.4	962.2	
1097	HLA-B*40:01	HLA-B*40:06	HLA-B*40:01:02	HLA-B*40:06:01:01	2642.2	1822.5	
1110	HLA-B*40:01	HLA-B*51:01	HLA-B*40:01:02	HLA-B*51:01:01	1277.1	1156.9	
1111	HLA-B*54:01	HLA-B*40:03	HLA-B*54:01:01	HLA-B*40:03	831.3	943.7	
1116	HLA-B*52:01	HLA-B*07:02	HLA-B*52:01:01:02	HLA-B*07:02:01	981.1	1535.6	
1117	HLA-B*52:01	HLA-B*51:01	HLA-B*52:01:01:02	HLA-B*51:01:01	5900.6	6084.9	Only 3 heterozygous SNVs
1119	HLA-B*35:01	HLA-B*07:02	HLA-B*35:01:01:02	HLA-B*07:02:01	921.3	1370.1	
1129	HLA-B*59:01	HLA-B*40:02	HLA-B*59:01:01:01	HLA-B*40:02:01	1472.9	2178.5	
1131	HLA-B*44:03	HLA-B*52:01	HLA-B*44:03:01	HLA-B*52:01:01:02	1519.9	1456.8	
1142	HLA-B*40:02	HLA-B*39:04	HLA-B*40:02:01	HLA-B*39:04	1253	886.5	
1144	HLA-B*44:03	HLA-B*15:11	HLA-B*44:03:01	HLA-B*15:11:01	1061.7	993.2	
1149	HLA-B*44:03	HLA-B*52:01	HLA-B*44:03:01	HLA-B*52:01:01:02	719.6	633.6	
1154	HLA-B*59:01	HLA-B*40:02	HLA-B*59;01:01:01	HLA-B*40:02:01	146.2	171.8	
1157	HLA-B*35:01	HLA-B*48:01	HLA-B*35:01:01:02	HLA-B*48:01:01	911	1077.3	
1160	HLA-B*59:01	HLA-B*54:01	HLA-B*59:01:01:02	HLA-B*54:01:01	129.8	166.8	
1161	HLA-B*55:02	HLA-B*51:01	HLA-B*55:02:01	HLA-B*51:01:01	402.5	366.6	
1163	HLA-B*56:01	HLA-B*46:01	HLA-B*56:01:01	HLA-B*46:01:01	2048.1	2276.5	
1165	HLA-B*15:01	HLA-B*07:02	HLA-B*15:01:01:01	HLA-B*07:02:01	688.1	907.3	
1167	HLA-B*40:06	HLA-B*52:01	HLA-B*40:06:01:01	HLA-B*52:01:01:02	1486.7	4508.1	
1169	HLA-B*54:01	HLA-B*54:01	HLA-B*54:01:01	HLA-B*54:01:01	7086.1		Homozygous
1171	HLA-B*54:01	HLA-B*44:03	HLA-B*54:01:01	HLA-B*44:03:01	1586.6	1833.9	
1175	HLA-B*15:11	HLA-B*15:01	HLA-B*15:11:01	HLA-B*HLA-B*15:01::01:01	5717.6	5693	Only 3 heterozygous SNVs
1182	HLA-B*55:02	HLA-B*51:01	HLA-B*55:02:01	HLA-B*51:01:01	1156.6	1451.5	
1183	HLA-B*54:01	HLA-B*35:01	HLA-B*54:01:01	HLA-B*35:01:01	1413.5	1537.7	
1184	HLA-B*52:01	HLA-B*52:01	HLA-B*52:01:01:02	HLA-B*52:01:01:02	6701.3		Homozygous
1199	HLA-B*51:01	HLA-B*51:01	HLA-B*51:01:01	HLA-B*51:01:01	5229.1		Homozygous
1201	HLA-B*44:03	HLA-B*52:01	HLA-B*44:03:01	HLA-B*52:01:01:02	1990.6	1665.9	
1203	HLA-B*54:01	HLA-B*52:01	HLA-B*54:01:01	HLA-B*52:01:01:02	1246.4	1313.2	
1217	HLA-B*40:01	HLA-B*46:01	HLA-B*40:01:02	HLA-B*46:01:01	2225.1	1882.2	
1224	HLA-B*40:02	HLA-B*40:03	HLA-B*40:02:01	HLA-B*40:03	6306.5	6286.4	Only 3 heterozygous SNVs
1225	HLA-B*40:01	HLA-B*44:03	HLA-B*40:01:02	HLA-B*44:03:01	1554	1399.6	
1229	HLA-B*52:01	HLA-B*52:01	HLA-B*52:01:01:02	HLA-B*52:01:01:02	6748.5		Homozygous
1234	HLA-B*44:03	HLA-B*40:06	HLA-B*44:03:01	HLA-B*40:06:01:01	2490.3	1715.9	
1236	HLA-B*54:01	HLA-B*40:06	HLA-B*54:01:01	HLA-B*40:06:01:01	1132.4	1244.4	
1238	HLA-B*40:02	HLA-B*67:01	HLA-B*40:02:01	HLA-B*67:01:01	2040.9	1691	
1239	HLA-B*40:03	HLA-B*51:01	HLA-B*40:03	HLA-B*51:01:01	1787	1789.8	
1250	HLA-B*44:03	HLA-B*51:01	HLA-B*44:03:01	HLA-B*51:01:01	357.7	317.8	
1259	HLA-B*40:02	HLA-B*07:02	HLA-B*40:02:01	HLA-B*07:02:01	1346.9	1934.4	
1260	HLA-B*40:06	HLA-B*35:01	HLA-B*40:06:01:01	HLA-B*35:01:01:02	641.9	680.8	
1265	HLA-B*40:01	HLA-B*40:06	HLA-B*40:01:02	HLA-B*40:06:01:01	2226.2	1691.7	

*Phase defined sequencing pipeline.

After obtaining phase-defined HLA gene sequences for 192 haplotype sequences, we attempted to assign HLA allele numbers to these sequences by searching for known allele sequences in the IMGT/HLA database. We used phased *HLA-B* haplotype sequences that spanned all of the intronic and exonic regions as queries against genomic and CDS sequences in the database. The determined *HLA-B* allele calls in all samples were consistent with PCR-SSO and Omixon Target, although the PCR-SSO determined HLA allele numbers were limited to a 4-digit resolution.

## Conclusions

We established a simple, high-throughput, high-resolution, and high-fidelity HLA sequencing and genotyping method, as a combination of the Nextera kit and our new BeNUS method. We successfully applied our method for *HLA-B* sequencing in 96 samples, without dropouts. By developing BeNUS, it becomes feasible to construct multi-libraries without agarose gel size selection and DNA density control of each library. This method would be greatly advantageous for clinical applications that require a user-friendly and cost-effective protocol, with high throughput and accuracy. Our protocols open a way to prepare NGS libraries for large-scale HLA gene sequencing and typing using an automated system.

## Methods

### Subjects

A total of 96 unrelated healthy Japanese control subjects were recruited at the Health Evaluation and Promotion Center of Tokai University Hospital. All subjects gave written informed consent for the study. Ethical approvals for this study protocol were obtained from the IRBs of National Institute of Genetics and Tokai University School of Medicine.

### DNA samples

DNA samples were extracted from peripheral blood using a DNA extraction kit Genomix (Biologica, Nagoya, Japan) using the manufacturer’s instructions.

### HLA genotyping with PCR-SSO method

We genotyped *HLA-B* using the Luminex assay system and HLA typing kits (WAKFlow HLA Typing kits, Wakunaga, Osaka, Japan or LABType SSO, One Lambda, Canoga Park, CA, USA).

### Library preparation

*HLA-B* was amplified using locus-specific primers by long-range PCR [1]. Each amplification reaction contained 20 ng of genomic DNA, 0.25 unit of PrimeSTAR® GXL DNA polymerase (TAKARA BIO Inc., Shiga, Japan), 1× PrimeSTAR® GXL buffer (Mg^2^+ concentration 1 mM), 0.2 mM of each dNTP, and 0.2 μM of each primer in a 10 μl reaction volume. Cycling parameters were as follows: initial denaturation of 94°C for 2 min followed by 30 cycles of 98°C for 10 s, 60°C for 15 s, and 68°C for 5 min. Each PCR product concentration was measured with a Qubit dsDNA BR Assay Kit (Life Technologies, Carlsbad, CA, USA). PCR products were subjected to library preparation with a Nextera DNA Sample Preparation Kit (Illumina, San Diego, CA, USA) and a KAPA Library Amplification kit (Kapa Biosystems, Inc., Wilmington, MA, USA). The KAPA kit was used for library amplification because of its advantage of coverage depth in high-GC-content regions during library amplification (Additional file 5: Figure S5). Each sample was dual indexed and normalized with modified AMPure XP beads (Beckman Coulter Inc., Brea, CA, USA) method, which include optimal size selection and normalization of DNA concentration (Figure 1).

### BeNUS for 96-well plate-based library

We prepared altered AMPure XP beads by resuspending the beads in half of the original volume of 20% polyethylene glycol 8000 (PEG) and 2.5 M NaCl solution. The resuspended beads were twice as concentrated as the standard AMPure XP beads (Additional file 6: Figure S6). The optimal volume ratio of altered beads to DNA solution was determined by relation between the PEG–NaCl concentration and the selected DNA fragment size (Additional file 6: Figure S6). For library size selection, 20 μl of resuspended altered beads was added to 50 μl of diluted library (5 μl of PCR product and 45 μl of water), mixed well by pipetting at least 10 times, and incubated for 5 min at room temperature. The tube was placed on the NGS MagnaStand (NIPPON Genetics, Tokyo, Japan) to separate the beads from the supernatant. After separation, the supernatant was carefully transferred to a new tube. Five μl of the altered beads were added to the supernatant, mixed, and incubated, and the beads were then separated from the supernatant on the same conditions. The supernatant containing unwanted DNA was carefully removed. One hundred μl of 80% ethanol was added to the tube for washing and the supernatant was carefully discarded after incubation and separation. The beads were air-dried for 10 min while the tube was on the magnetic stand with the lid open. The target library was eluted from the beads into 22 μl of water (Figure 1). Next, to normalize DNA concentration, altered beads, which were diluted 20 fold with 20% PEG and 2.5 M NaCl solution, were used to capture a certain amount of DNA (Additional file 2: Figure S2). Twenty μl of 20-fold diluted altered beads and 20 μl of isopropanol were added to the 20 μl of size-selected library, mixed well by pipetting at least 10 times, and incubated for 5 min at room temperature. The tube was placed on the magnetic stand to separate the beads from the supernatant. After the supernatant was discarded, 100 μl of 80% ethanol was added to the tube kept on the magnetic stand and incubated at room temperature for 30 s, and then the supernatant was carefully discarded. The beads were air-dried, and then 20 μl of water was added to elute the normalized libraries (Figure 1). The combination of fragment size selection and normalization of DNA amount results in an equalized DNA molar concentration among 96 libraries.

### Sequencing

Equal volumes of libraries were pooled and subjected to multiplex sequencing on the MiSeq sequencer (Illumina). The MiSeq flow cell of 2 × 300 bp paired-end reads resulted in 11.6 million read pairs corresponding to 6 Gbp of valid sequence data without adapter sequence.

### Determination of the *HLA-B*sequence

Sequence reads were distributed according to index information to assign samples. We used the phase-defined sequencing pipeline (http://p-galaxy.ddbj.nig.ac.jp) [1, 10], which include trimming low quality bases (Phred quality score < Q20), selection of only long (>200 bp) paired-end reads, alignment to reference sequence, SNVs and indel identification, and haplotype phasing. *HLA-B* sequence (UCSC hg19, chr6: 31,317,316 – 31,331,864, complement) was used as reference sequence. After phasing, two BAM files were created as phased *HLA-B* alignments. The IGV genome viewer [11] was used to visualize the alignment results. The consensus sequences in FASTQ or FASTA format were constructed for searching the IMGT/HLA (http://www.ebi.ac.uk/imgt/hla/) database to identify the HLA alleles. We used them as query for BLAT [12] search to known HLA allele sequences in the database as complete matches of genomic sequence or CDS sequence. For validation, *HLA-B* genotype calls were compared with the result of PCR-SSO and Omixon Target HLA Typing (Omixon Inc., Budapest, Hungary) [13, 14].

### Availability of supporting data

The data set associated with this project has been submitted to DDBJ Sequence Reads Archive (DRA accession number: DRA001289).

## Electronic supplementary material

Additional file 1: Figure S1: Fragment size selection focusing on the 500–1,000 bp size range. (PDF 98 KB)

Additional file 2: Figure S2: Association between number of beads and bound DNA for normalization of DNA concentration. (PDF 98 KB)

Additional file 3: Figure S3: Effect of DNA normalization as confirmed by BioAnalyzer. (PDF 134 KB)

Additional file 4: Figure S4: Example of partial phasing for a specific exon. (PDF 265 KB)

Additional file 5: Figure S5: KAPA Library Amplification kit showing high coverage in a high-GC-content region. (PDF 313 KB)

Additional file 6: Figure S6: Method for preparation of beads and optimal bead volume in the DNA solution. (PDF 132 KB)

## References

[1] Hosomichi K, Jinam TA, Mitsunaga S, Nakaoka H, Inoue I (2013). Phase-defined complete sequencing of the HLA genes by next-generation sequencing. BMC Genomics.

[2] Bentley G, Higuchi R, Hoglund B, Goodridge D, Sayer D, Trachtenberg EA, Erlich HA (2009). High-resolution, high-throughput HLA genotyping by next-generation sequencing. Tissue Antigens.

[3] Lind C, Ferriola D, Mackiewicz K, Heron S, Rogers M, Slavich L, Walker R, Hsiao T, McLaughlin L, D'Arcy M, Gai X, Goodridge D, Sayer D, Monos D (2010). Next-generation sequencing: the solution for high-resolution, unambiguous human leukocyte antigen typing. Hum Immunol.

[4] Erlich RL, Jia X, Anderson S, Banks E, Gao X, Carrington M, Gupta N, DePristo MA, Henn MR, Lennon NJ, de Bakker PI (2011). Next-generation sequencing for HLA typing of class I loci. BMC Genomics.

[5] Wang C, Krishnakumar S, Wilhelmy J, Babrzadeh F, Stepanyan L, Su LF, Levinson D, Fernandez-ViÃ±a MA, Davis RW, Davis MM, Mindrinos M (2012). High-throughput, high-fidelity HLA genotyping with deep sequencing. Proc Natl Acad Sci U S A.

[6] Lank SM, Wiseman RW, Dudley DM, O’Connor DH (2010). A novel single cDNA amplicon pyrosequencing method for high-throughput, cost-effective sequence-based HLA class I genotyping. Hum Immunol.

[7] Lank SM, Golbach BA, Creager HM, Wiseman RW, Keskin DB, Reinherz EL, Brusic V, O'Connor DH (2012). Ultra-high resolution HLA genotyping and allele discovery by highly multiplexed cDNA amplicon pyrosequencing. BMC Genomics.

[8] Shiina T, Suzuki S, Ozaki Y, Taira H, Kikkawa E, Shigenari A, Oka A, Umemura T, Joshita S, Takahashi O, Hayashi Y, Paumen M, Katsuyama Y, Mitsunaga S, Ota M, Kulski JK, Inoko H (2012). Super high resolution for single molecule-sequence-based typing of classical HLA loci at the 8-digit level using next generation sequencers. Tissue Antigens.

[9] Quail MA, Smith M, Coupland P, Otto TD, Harris SR, Connor TR, Bertoni A, Swerdlow HP, Gu Y (2012). A tale of three next generation sequencing platforms: comparison of Ion Torrent, Pacific Biosciences and Illumina MiSeq sequencers. BMC Genomics.

[10] Nagasaki H, Mochizuki T, Kaminuma E, Watanabe S, Morizaki S, Kodama Y, Saruhashi S, Takagi T, Okubo K, Nakamura Y (2013). DDBJ read annotation pipeline: a cloud computing based pipeline for high-throughput analysis of next generation sequencing data. DNA Res.

[11] Thorvaldsdóttir H, Robinson JT, Mesirov JP (2013). Integrative Genomics Viewer (IGV): high-performance genomics data visualization and exploration. Brief Bioinform.

[12] Kent WJ (2002). BLAT–the BLAST-like alignment tool. Genome Res.

[13] Major E, Rigó K, Hague T, Bérces A, Juhos S (2013). HLA typing from 1000 genomes whole genome and whole exome illumina data. PLoS One.

[14] Kulski JK, Suzuki S, Ozaki Y, Mitsunaga S, Inoko H, Shiina T, Xi Y (2014). In phase HLA genotyping by next generation sequencing - a comparison between two massively parallel sequencing bench-top systems, the Roche GS Junior and ion torrent PGM. HLA and Associated Important Diseases.

