# Supplementary figures and images for: A Bead-based Normalization for Uniform Sequencing depth (BeNUS) protocol for multi-samples sequencing exemplified by HLA-B
Source: BMC Genomics. 2014 Aug 4;15(1):645. doi: 10.1186/1471-2164-15-645 (PMC4133082; doi:10.1186/1471-2164-15-645)

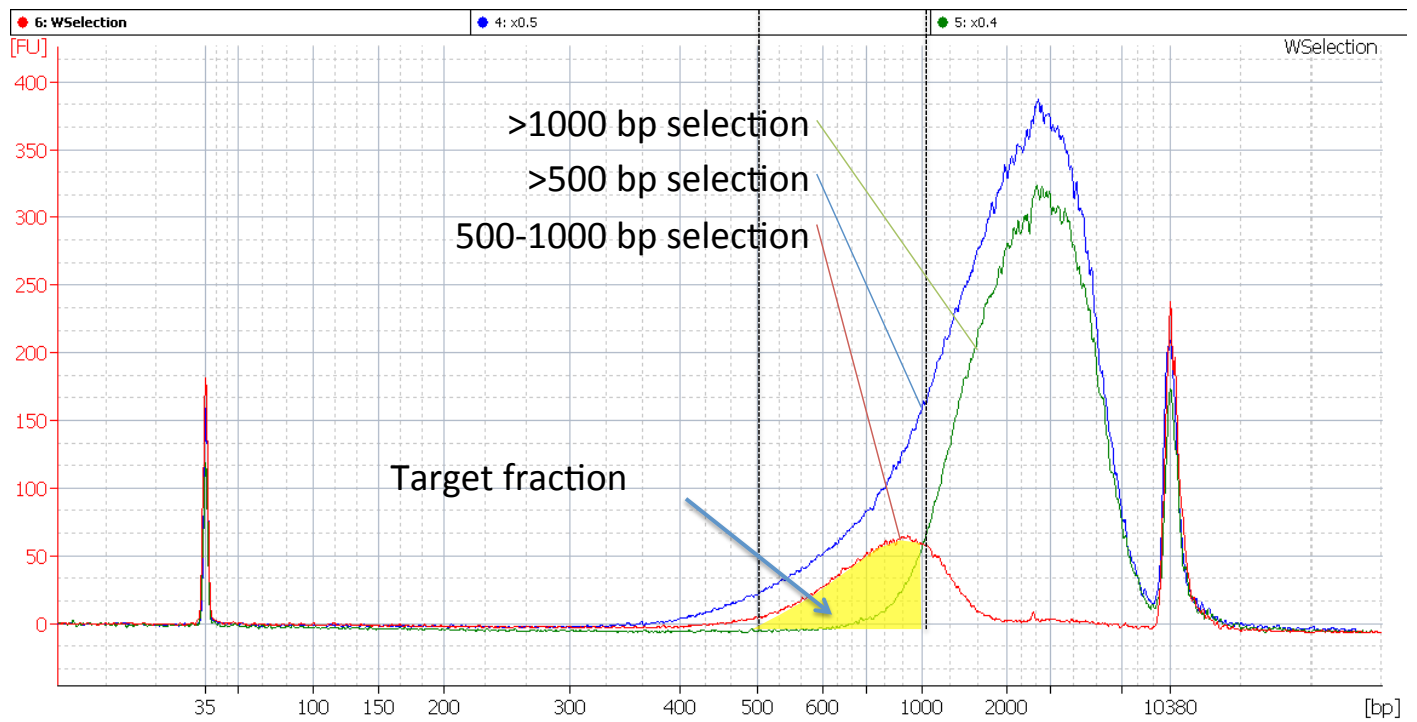

Supplementary figure 1 - Fragment size selection focusing on the 500 - 1,000 bp size range

Supplement: Supplementary file 1 — Additional file 1: Figure S1: Fragment size selection focusing on the 500–1,000 bp size range. (PDF 98 KB) [file 12864_2014_6340_MOESM1_ESM.pdf]

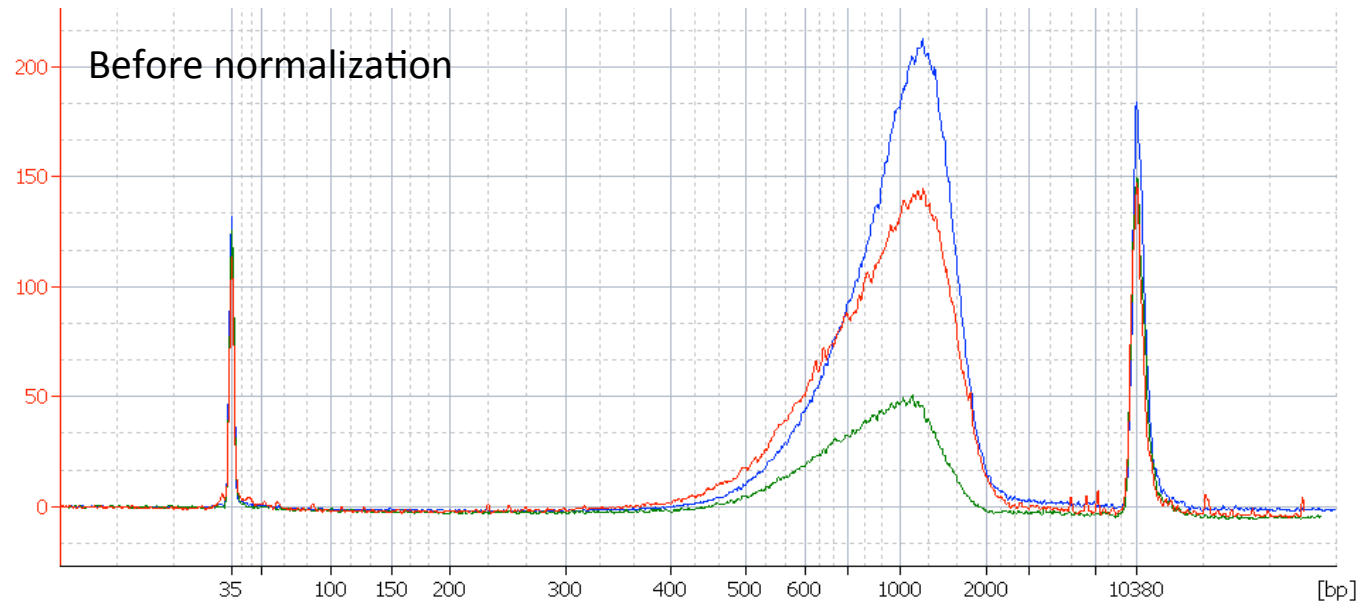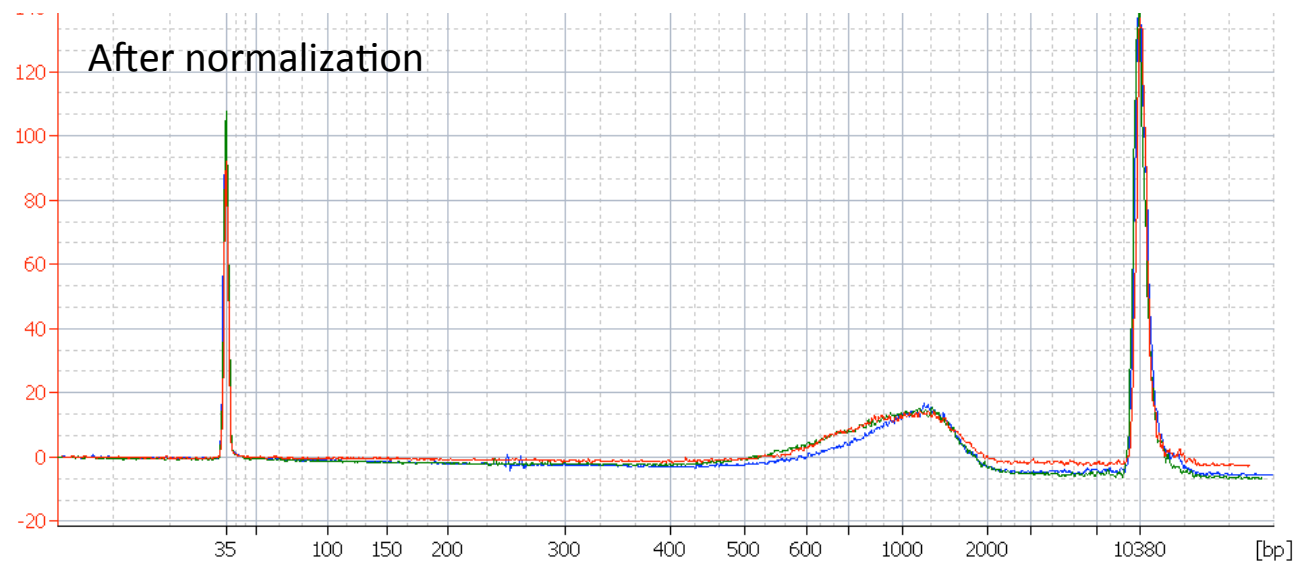

Supplementary figure 3 - Effect of DNA normalization as confirmed by BioAnalyzer

Supplement: Supplementary file 3 — Additional file 3: Figure S3: Effect of DNA normalization as confirmed by BioAnalyzer. (PDF 134 KB) [file 12864_2014_6340_MOESM3_ESM.pdf]

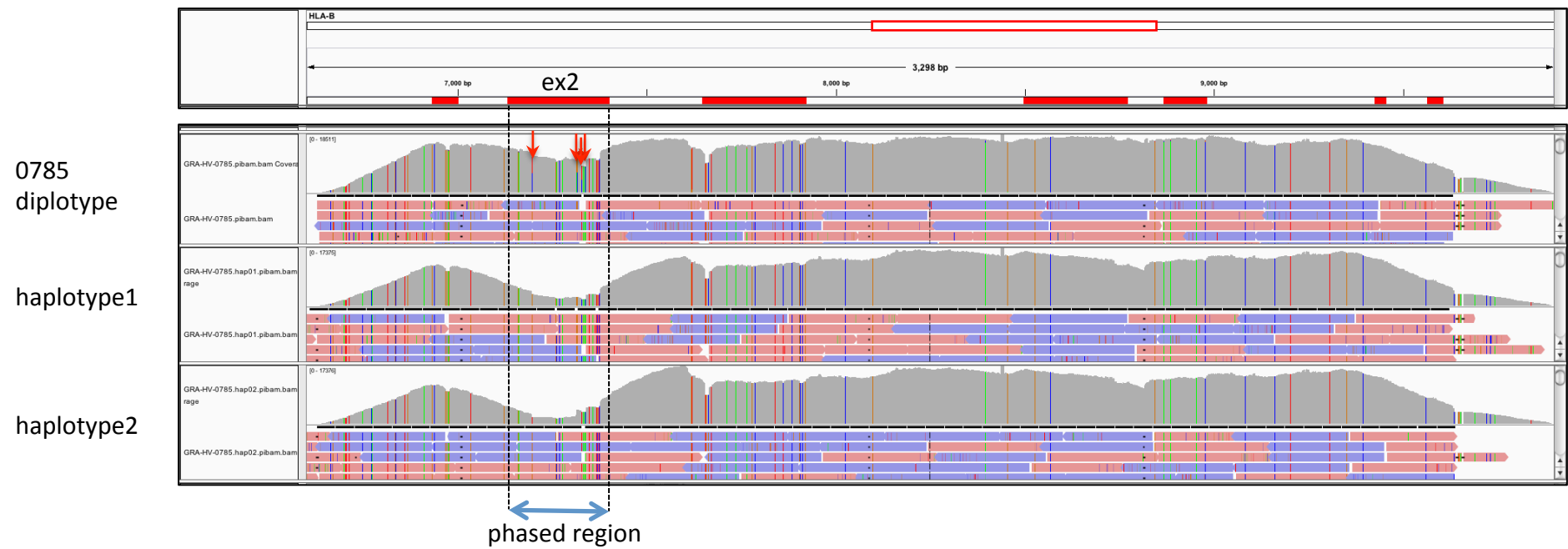

Supplementary figure 4 - Example of partial phasing for only a specific exon

Supplement: Supplementary file 4 — Additional file 4: Figure S4: Example of partial phasing for a specific exon. (PDF 265 KB) [file 12864_2014_6340_MOESM4_ESM.pdf]

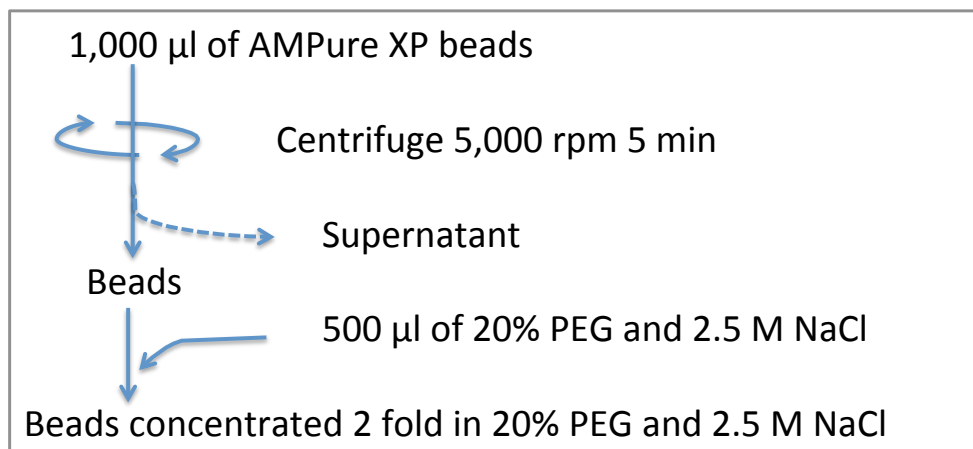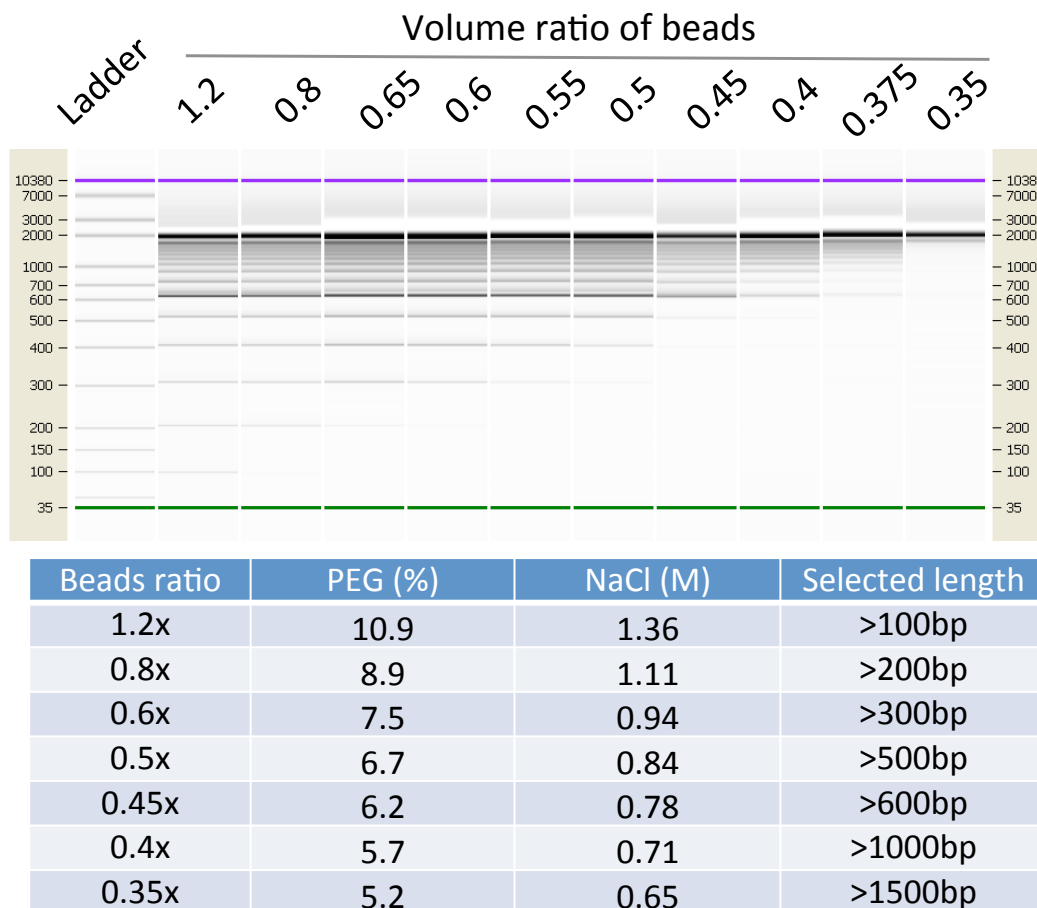

Supplementary figure 6 - Altered AMPure XP beads and optimal bead volume in the DNA solution

Supplement: Supplementary file 6 — Additional file 6: Figure S6: Method for preparation of beads and optimal bead volume in the DNA solution. (PDF 132 KB) [file 12864_2014_6340_MOESM6_ESM.pdf]
