# Supplementary material for: A Bead-based Normalization for Uniform Sequencing depth (BeNUS) protocol for multi-samples sequencing exemplified by HLA-B
Source: BMC Genomics. 2014 Aug 4;15(1):645. doi: 10.1186/1471-2164-15-645 (PMC4133082; doi:10.1186/1471-2164-15-645)
Supplement: Supplementary file 2 — Additional file 2: Figure S2: Association between number of beads and bound DNA for normalization of DNA concentration. (PDF 98 KB) [file 12864_2014_6340_MOESM2_ESM.pdf]

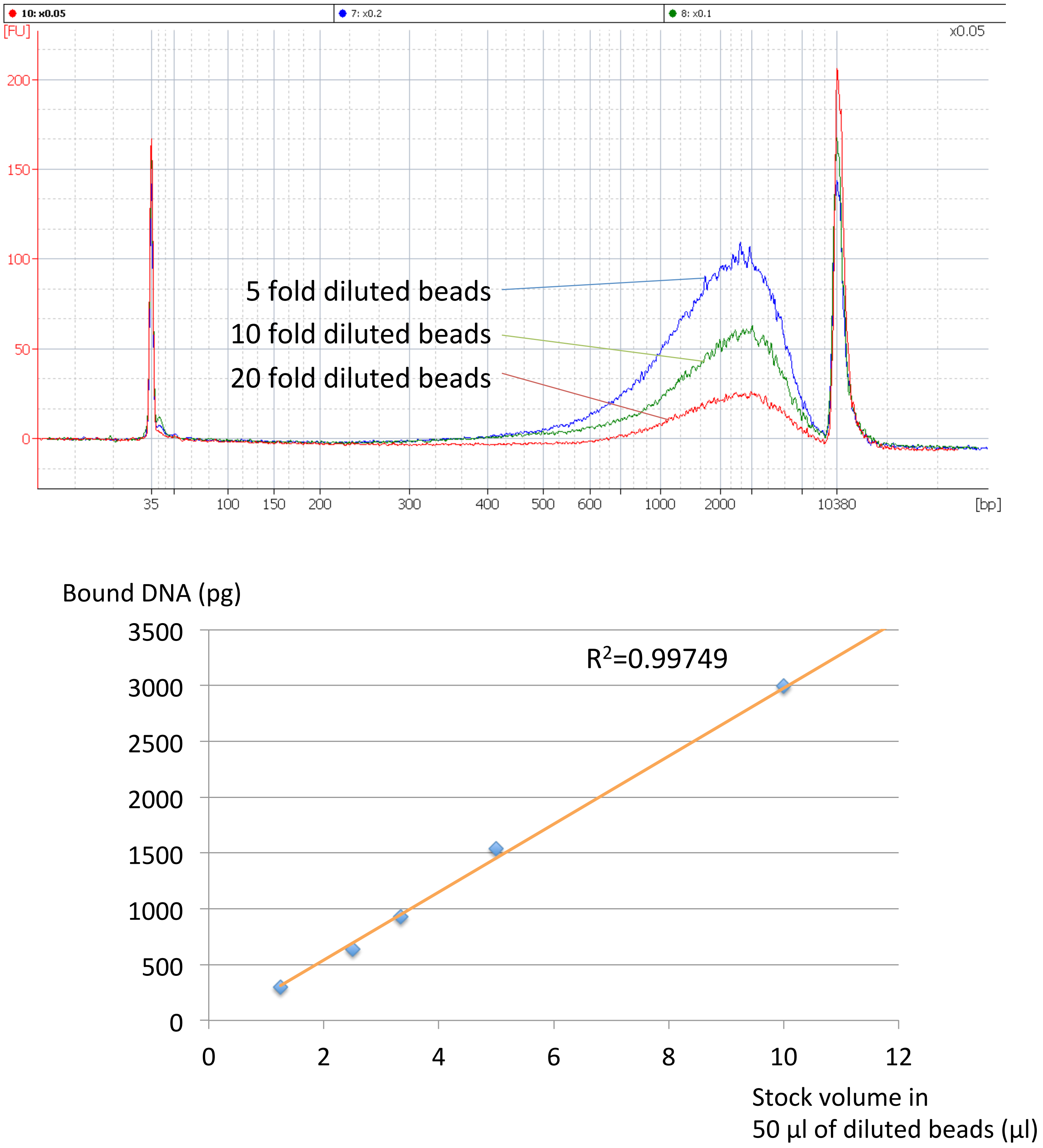

Supplementary figure 2 - Association between number of beads and bound DNA for normalization of DNA concentration
